# Radiation dose does not influence anastomotic complications in patients with esophageal cancer treated with neoadjuvant chemoradiation and transhiatal esophagectomy

**DOI:** 10.1186/s13014-015-0361-4

**Published:** 2015-03-06

**Authors:** Marijn Koëter, Maurice JC van der Sangen, Coen W Hurkmans, Misha DP Luyer, Harm JT Rutten, Grard AP Nieuwenhuijzen

**Affiliations:** Department of Surgery, Catharina Hospital Eindhoven, Michelangelolaan 2, 5623 EJ Eindhoven, The Netherlands; Department of Radiation Oncology, Catharina Hospital Eindhoven, Eindhoven, The Netherlands

**Keywords:** Radiation dose, Anastomotic complications, Esophageal cancer

## Abstract

**Background:**

Neoadjuvant chemoradiation might increase anastomotic leakage and stenosis in patients with esophageal cancer treated with neoadjuvant chemoradiation and esophagectomy. The aim of this study was to determine the influence of radiation dose on the incidence of leakage and stenosis.

**Methods:**

Fifty-three patients with esophageal cancer received neoadjuvant chemoradiation (23 × 1.8 Gy) (combined with Paclitaxel and Carboplatin) followed by a transhiatal esophagectomy between 2009 and 2011. On planning CT, the future anastomotic region was determined and the mean radiation dose, V20, V25, V30, V35 and V40 were calculated. Logistic regression analysis was conducted to examine determinants of anastomotic leakage and stenosis.

**Results:**

Anastomotic leaks occurred in 13 of 53 patients (25.5%) and anastomotic stenosis occurred in 24 of 53 patients (45.3%). Median follow-up was 20 months. Logistic regression analysis showed that mean dose, V20-V40, age, co-morbidity, method of anastomosis, operating time and interval between last radiotherapy treatment and surgery were not predictors of anastomotic leakage and stenosis.

**Conclusions:**

A radiation dose of 23 × 1.8 Gy on the future anastomotic region has no influence on the occurrence of anastomotic leakage and stenosis in patients with esophageal cancer treated with neoadjuvant chemoradiation followed by transhiatal esophagectomy.

## Background

Esophageal cancer is the eighth most commonly diagnosed type of cancer worldwide and it is the sixth leading cause of cancer deaths [1]. The incidence of esophageal carcinoma in the Netherlands, especially adenocarcinoma, has rapidly risen from 1731 new cases in 2000 to 2499 in 2010 [2]. According to the current Dutch guidelines, the preferred curative treatment for non-metastatic disease is neoadjuvant chemoradiation followed by transhiatal or transthoracic esophagectomy [3]. Patients with an irresectable tumor, or patients who are too vulnerable for surgery are often proposed for definitive chemoradiation which show encouraging results [4,5].

Surgical treatment has an acceptable mortality in high volume centres, but high rates of post-operative morbidity have still been described [6-9]. However, pulmonary complications and anastomotic complications like leakage and stenosis are still common [7-9]. The incidence of anastomotic leakage reported in the literature ranges from 5.7% to 41% [6-16]. Incidence rates of anastomotic stenosis are even higher ranging from 21.8% to 44% [6,10-12].

Factors like co-morbidity, nutrition status, anastomotic location, anastomotic technique and blood loss during surgery are hypothesised to be related to the development of anastomotic leakage and stenosis [10,17].

Neoadjuvant chemoradiation might also play a role in developing anastomotic complications. Studies comparing neoadjuvant chemoradiation followed by surgery with surgery alone showed conflicting results with respect to the risk of anastomotic leakage and stenosis due to the neoadjuvant treatment [8,11,15,16,18]. However, these studies incorporated heterogeneous patient groups, radiation fields and anastomotic locations. A recent study showed that in patients receiving neoadjuvant chemoradiation median radiation dose to the gastric fundus was an independent predictor for early anastomotic complications in patients with an Ivor-Lewis esophagectomy [19]. However in an intrathoracic anastomosis the region below the gastric fundus rather than the gastric fundus itself is used for the anastomosis because a shorter gastric conduit is needed when compared with a cervical anastomosis, raising the question of whether or not other factors are responsible for the observed difference. When compared with an Ivor-Lewis esophagectomy, in patients receiving a transhiatal resection and a cervical anastomosis, a larger part of the irradiated gastric fundus is used for the anastomosis. Hence, the aim of our study was to determine the influence of radiation dose on the incidence of anastomotic complications (leakage and stenosis) in a more homogeneous patient group with distal esophageal or gastro-esophageal junction cancer undergoing neoadjuvant chemoradiation followed by a transhiatal esophagectomy and cervical anastomosis. In all of these patients the fundus of the stomach was irradiated to a varying degree.

## Methods

### Study population

Between 2009 and 2011 we included 53 consecutive patients with distal esophageal cancer (C15.5) or gastro-esophageal junction cancer (C16.0), who received neoadjuvant chemoradiation followed by an open or laparoscopic transhiatal esophagectomy with a left cervical anastomosis. Median follow-up duration was 20 months (range 0.2-25). All patients had histologically proven adenocarcinoma or squamous cell carcinoma with no evidence of distant metastases (cT1-3, N0-3, M0; TNM 7) [20]. Cancer staging included clinical examination, esophago-gastroscopy with biopsies, endoscopic ultrasonography (EUS), external ultrasonography of the cervical region, computed tomography (CT) of the chest and abdomen and a positron emission tomography fused with CT (PET- CT). This research is reviewed by the local medical ethics committee but the Dutch Medical Research (Human Subjects) Act is not applicable to this study.

### Surgery

Surgical treatment consisted of a laparoscopic or open transhiatal esophagectomy with gastric tube interponate [18]. A left cervical anastomosis was performed (at surgeon’s preference) end-to-end with hand-sewn continuous or interrupted sutures or side-to-side with a stapling device (Collard anastomosis [21]).

### Neoadjuvant chemoradiation regimen

**Figure 1:**
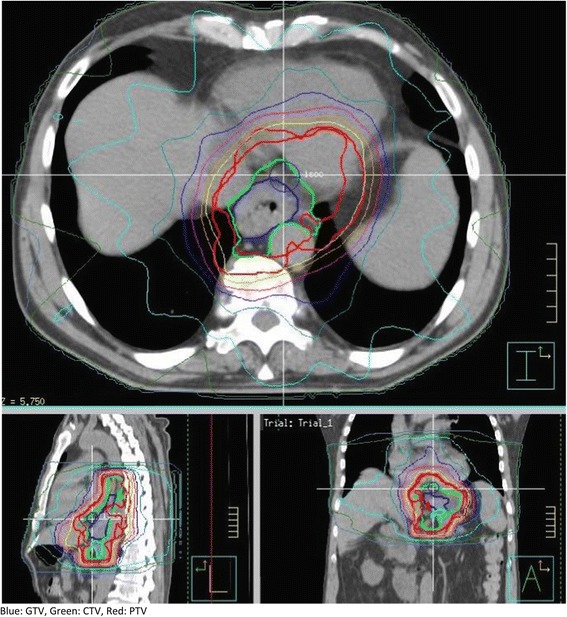
**Example pictures of dose distribution.**

The neoadjuvant regimen consisted of Three-Dimensional Conformal Radiotherapy (3D-CRT) to a total dose of 41.4 Gy (23 fractions of 1.8 Gy, 5 fractions a week) combined with Paclitaxel (50 mg/m^2^) and Carboplatin (AUC = 2) administered by intravenous infusion on days 1, 8, 15, 22 and 29. The Gross Tumor Volume (GTV) included all visible tumor and pathologically enlarged lymph nodes (determined by CT, PET-CT or EUS). The Clinical Target Volume (CTV) was defined by the GTV (node and tumor) plus the area of regional lymph nodes up to at least 3 cm in cranial and caudal extension of the esophagus from the tumor GTV. To ensure adequate margins around the macroscopic tumor, a minimum CTV-GTV margin of 0.5 cm was required. For distal tumors, the caudal margin should follow the wall of esophagus and cardia. The margin in the direction of the wall of the cardia was limited to 2 cm. The Planning Target Volume (PTV) consisted of the CTV plus a margin of 1 cm in all directions (Figure 1). These margins were chosen as these are the margins we use in clinical practice. We realise that these margins should preferably be patient specific and dependent on, for example, the individual tumor motion. Because of these margins, the fundus in all patients with distal or junction tumors was irradiated to a varying degree.

### Calculation of RT dose to the anastomotic region of interest

**Figure 2:**
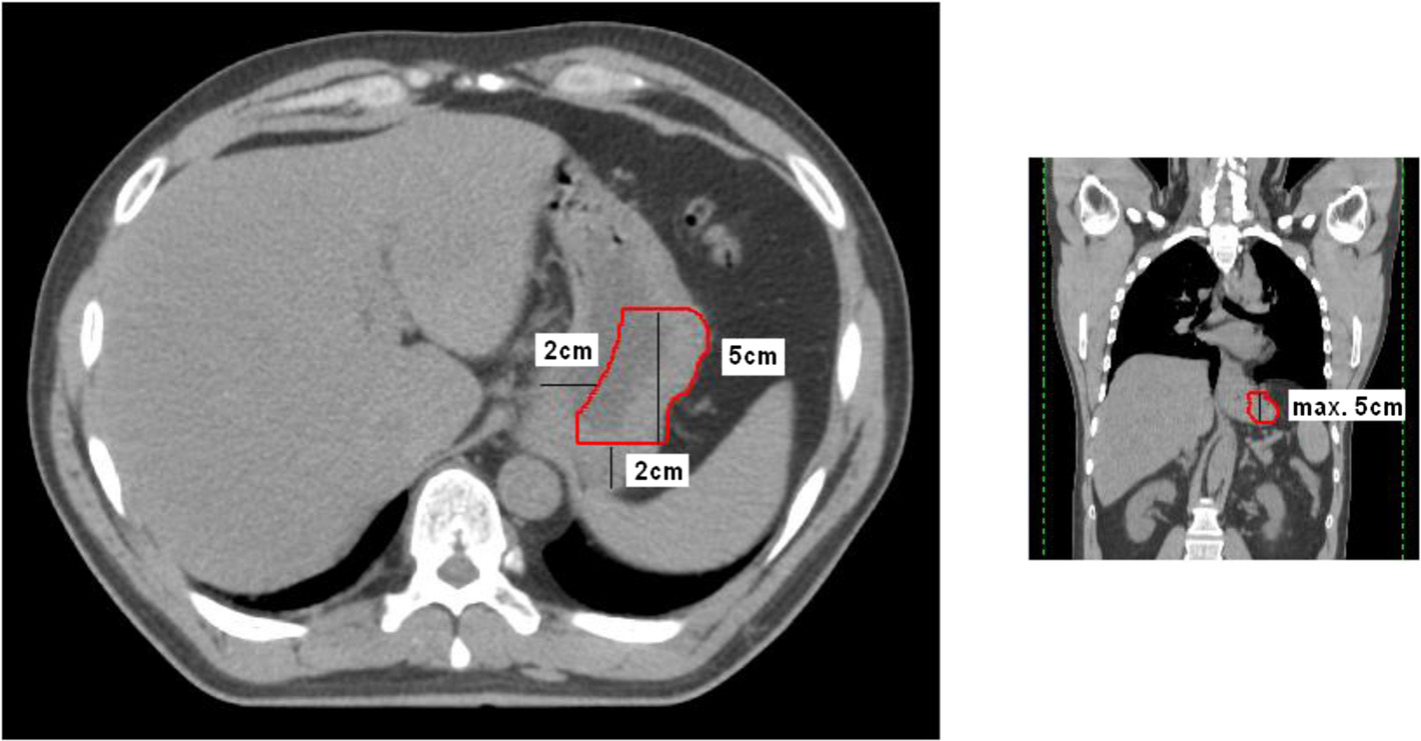
**Example of a planning CT in which the future anastomotic region is drawn.**

The future anastomotic region was retrospectively determined on the preoperative planning CT using the Philips Pinnacle treatment planning system version 9.0. CT slice thickness and separation were 3 mm. The most proximal part of the stomach was determined. From that point, a 5 cm distal (coronal plane) vertical line was drawn. On the transversal plane the distal margin at 7 cm was drawn. We used a 2 cm margin from the lesser curvature and a 2 cm margin from the most proximal part of the stomach (Figure 2). These margins are determined after consultation of the operating surgeon. The future anastomotic region in all patients was determined by the first author.

From this future anastomotic region we calculated the following parameters: volume, mean dose, V20, V25, V30, V35 and V40 (percentage irradiated volume receiving more than respectively 20, 25, 30, 35 and 40 Gy). In order to quantify the effect of a larger CTV-PTV margin on the parameters we repeated the analysis with an expansion of 0.5 cm in all directions (anastomotic region volume + 0.5 cm).

### Classification of leakage and stenosis

Anastomotic leakage was defined as any clinical evidence of leakage of salivary fluid in the cervical region, gastric conduit necrosis or evidence of anastomotic leakage on CT or with esophago-gastroscopy (CTCAE grade 1–5) [22]. Anastomotic stenosis was defined as dysphagia for which one or more endoscopic dilatation(s) of the anastomosis was needed.

### Statistical analysis

Differences in patient, tumor and dose characteristics between patients with or without anastomotic leakage or anastomotic stenosis were compared using the Mann–Whitney *U* test and the chi-square test. Univariable and multivariable logistic regression analyses were conducted to evaluate the potential risk factors for developing anastomotic leakage or stenosis. All analyses were performed using Statistical Package for Social Sciences version 19.0 (SPSS Inc., Chicago, IL, USA). All reported p-values below 0.05 were considered statistically significant.

## Results

All 53 patients completed the neoadjuvant chemoradiation regimen followed by transhiatal esophagectomy after a therapy free interval of 4–18 weeks, with a median of 9 weeks. The mean irradiation dose to the anastomotic region was 30.3 Gy [range 6–42], with a mean volume of 48.2 cm^3^ [range 21–92]. Postoperatively, 13 of 53 patients (25.5%) developed an anastomotic leak and six patients (11.3%) needed a surgical re-intervention; of these, 4 patients (7.5%) required re-intervention because of severe anastomotic problems. Two patients needed thoracic drainage for thoracic empyema. In three patients, drainage of a cervical abscess was performed. One patient needed re-intervention for an abdominal dehiscence. Two patients (3.8%) died in hospital: one patient died because of Acute Respiratory Distress Syndrome and another of myocardial infarction. To date, 15 patients (28.3%) have died during follow-up, with median follow-up duration of 20 months. Of these patients, 10 died because of cancer recurrence, 3 patients died from a non-disease-related cause and 2 patients had an unknown cause of dead.

**Figure 3:**
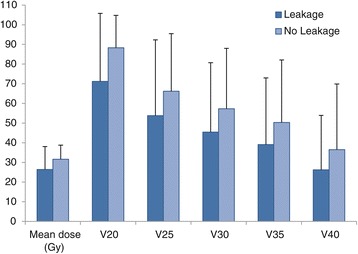
**Percentage of patients with and without leakage as a function of DVH parameters.** Error bars represent 1 SD.

Patients with an anastomotic leakage were hospitalised significantly longer than patients without anastomotic leakage (22 vs. 13 days, p = 0.001). Between the groups with or without anastomotic leakage no significant differences in age, gender, BMI, co-morbidity, ASA classification, histology, type of operation, duration of the procedure, method of anastomosis, anastomotic region volume, mean dose and time between the end of the neoadjuvant treatment and surgery were observed (Table 1). Comparable results on leakage rate were observed using the anastomotic region volume + 0.5 cm. In Figure 3 we depicted the percentage of patients with and without leakage as a function of DVH parameters.

**Table 1:** **Characteristics of patients with or without anastomotic leakage, treated with neo-adjuvant chemoradiation followed by transhiatal esophagectomy**

	**Anastomotic leakage**		
	**Yes (n = 13)**	**No (n = 40)**	**p-value**
Mean age	62.4 [41–77]	63.8 [41–82]	0.83
Gender:			
Male (n = 49)	13 (27%)	36 (73%)	
Female (n = 4)	0 (0%)	4 (100%)	0.56
BMI	27.5 [21–41]	26.4 [19–35]	0.65
Co-morbidity:			
0 (n = 13)	3 (23%)	10 (77%)	
1 (n = 18)	4 (22%)	14 (78%)	
2 or more (n = 22)	6 (27%)	16 (73%)	0.93
ASA classification:			
I (n = 8)	3 (38%)	5 (63%)	
II (n = 41)	9 (22%)	32 (78%)	
III (n = 4)	1 (25%)	3 (75%)	0.65
Histologic type:			
Adenocarcinoma (n = 49)	12 (24%)	37 (76%)	
Squamous cell carcinoma (n = 4)	1 (25%)	3 (75%)	0.98
Mean volume (cm^3^)	53.1 [33–71]	46.6 [21–92]	0.10
Mean dose (Gy)	26.4 [6–41]	31.6 [15–42]	0.16
V20	71.2%	88.3%	0.54
V25	53.8%	66.2%	0.27
V30	45.4%	57.3%	0.24
V35	39.1%	50.3%	0.24
V40	26.2%	36.5%	0.32
OR time (min)	172 [118–329]	187 [134–292]	0.09
Resection type:			
Open (n = 21)	7 (33%)	14 (67%)	
Laparoscopic (n = 32)	6 (19%)	26 (81%)	0.23
Method of anastomosis:			
End-to-end continuous (n = 33)	6 (18%)	27 (82%)	
End-to-end interrupted (n = 11)	4 (36%)	7 (64%)	
Side-to -side stapler (n = 9)	3 (33%)	6 (67%)	0.38
Interval last RT– surgery (days)	70 [40–127]	69 [31–121]	0.87
Hospital stay (days)	22 [13–71]	13 [6–35]	0.001

**Figure 4:**
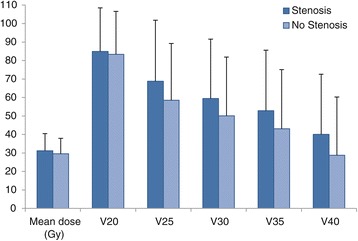
**Percentage of patients with and without stenosis as a function of DVH parameters.** Error bars represent 1 SD.

Clinically, anastomotic stenosis occurred in 24 of 53 patients (45.3%). Between the groups with or without anastomotic stenosis, no significant differences in age, gender, BMI, co-morbidity, ASA classification, histology, type of operation, operating time, method of anastomosis, anastomotic region volume, mean dose and time between the end of the neoadjuvant treatment and surgery were observed (Table 2). Comparable results on stenosis rate were observed using the anastomotic region volume +0.5 cm. In Figure 4 we depicted the percentage of patients with and without stenosis as a function of DVH parameters.

**Table 2:** **Characteristics of patients with or without anastomotic stenosis, treated with neo-adjuvant chemoradiation followed by transhiatal esophagectomy**

	**Anastomotic stenosis**		
	**Yes (n = 24)**	**No (n = 29)**	**p-value**
Mean age	61.3 [41–78]	65.4 [48–82]	0.30
Gender:			
Male (n = 49)	21 (43%)	28 (57%)	
Female (n = 4)	3 (75%)	1 (25%)	0.32
BMI	26.6 [19–41]	26.7 [21–35]	0.68
Co-morbidity:			
0 (n = 13)	9 (69%)	4 (31%)	
1 (n = 18)	8 (44%)	10 (56%)	
2 or more (n = 22)	7 (32%)	15 (68%)	0.10
ASA classification:			
I (n = 8)	4 (50%)	4 (50%)	
II (n = 41)	19 (46%)	22 (54%)	
III (n = 4)	1 (25%)	3 (75%)	0.69
Histologic type:			
Adenocarcinoma (n = 49)	22 (45%)	27 (55%)	
Squamous cell carcinoma (n = 4)	2 (50%)	2 (50%)	0.84
Volume (cm^3^)	48.3 [29–81]	48.1 [21–92]	0.92
Mean dose (Gy)	31.2 [6–42]	29.6 [8–42]	0.38
V20	84.9%	83.4%	0.27
V25	68.8%	58.5%	0.24
V30	59.5%	50.1%	0.30
V35	52.9%	43.1%	0.28
V40	40.1%	28.8%	0.14
OR time (min)	184 [118–329]	182 [138–292]	0.75
Resection type:			
Open (n = 21)	11 (52%)	10 (48%)	
Laparoscopic (n = 32)	13 (41%)	19 (59%)	0.40
Method of anastomosis:			
End-to-end continuous (n = 33)	15 (45%)	18 (55%)	
End-to-end interrupted (n = 11)	7 (64%)	4 (36%)	
Side-to -side stapler (n = 9)	2 (22%)	7 (78%)	0.18
Interval last RT– surgery (days)	65 [37–118]	73 [31–127]	0.31
Hospital stay (days)	14 [6–35]	16 [6–71]	0.63
Anastomotic leakage (n = 13)	7 (54%)	6 (46%)	0.48

Univariable logistic regression analysis showed that mean radiation dose was a borderline significant predictor for anastomotic leakage. In addition univariable analysis also showed that patients with a high V20 percentage were less likely to develop anastomotic leakage. However, multivariable analysis showed that V20 percentage and mean dose to the proposed area of the anastomosis were not significant predictors for anastomotic leakage anymore. Again, all other factors like age, BMI, co-morbidity (including cardiovascular and pulmonary co-morbidity separately), histology, ASA classification, type of resection, operating time, method of anastomosis, mean dose, V25-V40 and interval between the end of the neoadjuvant treatment and surgery were not significant predictors for anastomotic leakage in our univariable analysis and were therefore not analysed in the multivariable analysis (Table 3). Furthermore, the same analyses for the anastomotic region volume + 0.5 cm had no influence on the results.

**Table 3:** **Predictors of anastomotic leakage in patients with distal esophageal or esophago-gastric junction cancer treated with neoadjuvant chemoradiation followed by transhiatal esophagectomy**

	**Univariable analysis**			**Multivariable analysis**		
	**OR**	**95% CI**	**p-value**	**OR**	**95% CI**	**p-value**
Age	0.99	0.93-1.05	0.63			
BMI	1.07	0.91-1.24	0.42			
Co-morbidity:						
0	0.80	0.16-3.94	0.78			
1	0.76	0.18-3.26	0.71			
2 or more	ref					
Cardiovascular co-morbidity:						
No	1.29	0.37-4.53	0.69			
Yes	ref					
Pulmonary co-morbidity:						
No	ref					
Yes	1.7	0.36-8.04	0.50			
ASA classification:						
I	2.13	0.43-10.68	0.36			
II	ref					
III	1.19	0.11-12.82	0.89			
Histology:						
Adenocarcinoma	ref					
Squamous cell carcinoma	0.98	0.10-10.83	0.98			
Mean dose	1.00	1.00-1.00	0.07	1.00	1.00-1.00	0.67
V20	0.97	0.96-1.00	0.03	0.96	0.90-1.02	0.21
V25	0.99	0.97-1.01	0.23			
V30	0.99	0.98-1.01	0.25			
V35	0.99	0.97-1.01	0.28			
V40	0.99	0.97-1.01	0.32			
OR time	0.99	0.97-1.01	0.25			
Resection type:						
Open	2.17	0.61-7.71	0.23			
Laparoscopic	ref					
Method of anastomosis:						
EE continuous	ref					
EE interrupted	2.57	0.57-11.69	0.22			
Side-to -side stapler	2.25	0.44-11.65	0.33			
Interval last RT- surgery	1.00	0.83-1.20	0.99			

Univariable analysis showed that age and interval between the end of the neoadjuvant treatment and surgery were borderline significant. Anastomotic stenosis developed significantly more often in patients without comorbidity compared with patients with > =1 comorbidity. However, multivariable analysis showed that factors such as age, co-morbidity and interval between radiotherapy dose and surgery were not significant predictors for anastomotic stenosis. All other factors like BMI, cardiovascular and pulmonary co-morbidity, histology, ASA classification, type of operation, operating time, method of anastomosis, mean dose, and V20-V40 were not significant predictors for anastomotic stenosis in our univariable analysis and were therefore not analysed in the multivariable analysis (Table 4). Again, the same analyses for the ROI + 0.5 cm had no influence on the results observed in our analysis.

**Table 4:** **Predictors of anastomotic stenosis in patients with distal esophageal or esophago-gastric junction cancer treated with neoadjuvant chemoradiation followed by transhiatal esophagectomy**

	**Univariable analysis**			**Multivariable analysis**		
	**OR**	**95% CI**	**p-value**	**OR**	**95% CI**	**p-value**
Age	0.96	0.90-1.01	0.14	0.99	0.92-1.06	0.66
BMI	0.99	0.87-1.14	0.92			
Co-morbidity:						
0	4.82	1.10-21.19	0.04	3.17	0.54-18.82	0.20
1	1.71	0.77-6.24	0.41	1.39	0.34-5.67	0.64
2 or more	ref					
Cardiovascular co-morbidity:						
No	2.73	0.89-8.33	0.78			
Yes	ref					
Pulmonary co-morbidity:						
No	ref					
Yes	0.96	0.23-4.06	0.96			
ASA classification:						
I	1.16	0.25-5.72	0.85			
II	ref					
III	0.39	0.04-4.03	0.43			
Histology:						
Adenocarcinoma	ref					
Squamous cell carcinoma	1.23	0.16-9.43	0.84			
Mean dose	1.00	1.00-1.00	0.50			
V20	1.00	0.98-1.03	0.81			
V25	1.01	0.99-1.03	0.24			
V30	1.01	0.99-1.03	0.28			
V35	1.01	0.99-1.03	0.27			
V40	1.01	0.99-1.03	0.21			
OR time	1.00	0.99-1.01	0.87			
Resection type:						
Open	1.61	0.53-4.88	0.40			
Laparoscopic	ref					
Method of anastomosis:						
EE continuous	ref					
EE interrupted	2.10	0.51-8.57	0.30			
Side-to -side stapler	0.34	0.06-1.90	0.22			
Interval last RT- surgery	0.88	0.74-1.04	0.14	0.92	0.77-1.11	0.39
Anastomotic leakage	1.58	0.45-5.55	0.48			

## Discussion

In this study we determined the influence of radiation dose in the future anastomotic region on developing anastomotic leakage and stenosis in patients with distal esophageal or esophago-gastric junction cancer (cT1-3, N0-3, M0) treated with neoadjuvant chemoradiation followed by transhiatal esophagectomy with gastric tube reconstruction and a left cervical anastomosis. Overall, 25.5% of patients developed anastomotic leakage and 45.3% developed anastomotic stenosis. Our study identified no significant predictors of anastomotic leakage and stenosis. In contrast with a recent study [19], radiation dose did not have a significant influence on developing anastomotic leakage and stenosis.

This study shows that variations in mean dose and V20 until V40 had no significant influence on developing anastomotic leakage. However, a recent study demonstrated that the mean radiation dose on the gastric fundus dose was an independent predictor for early anastomotic complications like leakage [19]. The recent study had a different neoadjuvant treatment regime (36 Gy in 20 fractions combined with 5-FU and cisplatin) and a different surgical procedure (Ivor-Lewis) compared with the current study. Although the authors did not describe their surgical procedure in detail, the region below the gastric fundus at the level of the watershed of the gastroepiploic vessels is most commonly used for the intra-thoracic anastomosis in the Ivor-Lewis procedure. In a cervical anastomosis, the gastric tube should be longer and the anastomosis reconstructed from the fundus region of the stomach. Hence, the radiation dose on the future anastomotic region could even be less than the dose suggested by the authors and other factors could potentially be more responsible for the observed effects than the irradiation on the gastric fundus itself. Therefore, it is even more remarkable that we did not observe a negative effect of irradiation to the fundus on anastomotic complications in our series. Furthermore, the authors did not have a clear definition of anastomotic leakage; in addition, their description of the determined anastomotic region was not clear and not reproducible.

In our study, variations in mean dose and V20-V40 showed no significant influence on developing anastomotic stenosis. This is in concordance with a study in which it was demonstrated that neoadjuvant chemoradiation was not a predictor for the incidence of benign anastomotic strictures. However, this study did show that neoadjuvant chemoradiation was an independent predictor for patients with refractory strictures (requiring >10 dilatations) [18]. Furthermore, a recent study showed a higher mean radiation dose in patients with anastomotic stenosis [19].

Comorbidity in our study did not have an influence on the development of anastomotic leakage or stenosis. This is in concordance with another study showing that major co-morbidity is not an independent predictor for early anastomotic complications like leakage [19]. Another study confirmed these results and showed that diabetes, chronic obstructive pulmonary disease and coronary artery disease had no influence on developing anastomotic leakage [8].

Anastomotic leakage rates vary between studies, with leakage rates between 5.7 and 41% [6-16]. This might be the result of the various definitions of anastomotic leakage. Some studies define leakage as clinical or radiological evidence of leakage. However, other definitions include only clinical leakage or anastomotic leakage requiring re-intervention.

In this study, anastomotic leakage was defined as every evidence of leakage (including all clinical and radiologic evidence of leakage), explaining the relatively high rate of anastomotic leakage (25.5%) in our study compared with others [6-15]. As summarised in Table 5, randomized controlled trials that have compared neo-adjuvant chemoradiation with subsequent surgery versus surgery alone show different results with regard to the incidence of anastomotic leakage and the influence of chemoradiation on leakage rate. The incidence of leakage rates in those RCTs ranged from 3% to 30% [16,23-30]. The influence of neo-adjuvant chemoradiation on leakage rate is often not statistically tested. Furthermore, the differences in leakage rate between patients receiving neo-adjuvant chemoradiation with surgery vs. surgery alone are small.

**Table 5:** **Anastomotic complications in randomised trials comparing neo-adjuvant chemoradiation and surgery with surgery alone**

**Anastomotic complications neo-adjuvant chemoradiation and surgery vs. surgery alone**										
**Study**	**Groups no**		**CRT scheme**		**Surgery type**	**Anastomotic location**	**Anastomotic leakage**		**Anastomotic stenosis**	
	**CRT + S**	**S**	**Chemo**	**RT**			**CRT + S**	**S**	**CRT + S**	**S**
Nygaard et al. 1992 [23]	34	38	Cisplatin	35.0 Gy (20 × 1.75)	TTE	Not mentioned	6%	3%	Not Mentioned	
Le Prise et al. 1993 [24]	41	45	Cisplatin 5-FU	20.0 Gy (10 × 2.0)	Not mentioned	Not mentioned	Not Mentioned		Not Mentioned	
Walsh et al. 1996 [25]	58	55	Cisplatin 5-FU	40.0 Gy (15 × 2.67)	TTE or THE	Cervical	3%	4%	Not Mentioned	
Bosset et al. 1997 [26]	143	139	Cisplatin	37.0 Gy (10 × 3.7)	TTE >80%	Not mentioned	Not Mentioned		Not Mentioned	
Lee et al. 2004 [27]	51	50	Cisplatin 5-FU	45.6 Gy (38 × 1.2)	TTE	Cervical	Not Mentioned		14%	17%
Urba et al. 2001 [28]	50	50	Cisplatin 5-FU Vinblastin	45.0 Gy (30 × 1.5)	THE	Cervical	15%	8%	Not Mentioned	
Burmeister et al. 2005 [29]	128	128	Cisplatin 5 FU	35.0 Gy (15 × 2.3)	TTE	Thoracic or Cervival	5%	5%	19%	24%
Tepper et al. 2008 [30]	30	26	Cisplatin 5-FU	50.4 Gy (28 × 1.8)	TTE or THE	Not mentioned	Not Mentioned		Not Mentioned	
Van Hagen et al. 2012 [16]	178	188	Carboplatin Paclitaxel	41.4 Gy (23 × 1.8)	TTE or THE	Cervical	22%	30%	Not Mentioned	

When comparing our leakage rate with the rate reported in a recent Dutch multicentre randomized trial studying the role of neo-adjuvant chemoradiation with the same regime as our study, our leakage rate appears to be comparable [16].

The influence of (chemo)radiation on anastomotic leakage has also been studied in other solid tumors like rectal cancer. A large randomized and a large retrospective study showed that neo-adjuvant chemoradiation did not influence anastomotic leakage rates following Total Mesorectal Excision in patients with rectal cancer [31,32]. Another large Dutch Multicentre RCT showed no difference in anastomotic leakage rates between patients who received short course neoadjuvant radiotherapy followed by surgery compared to surgery alone. However, they did observe significantly more post-operative wound complications in the radiotherapy group [33].

Anastomotic stenosis occurred in 45.3% of the patients; however, other studies have shown a lower incidence (21.8-41.7%) [6,11,12,18]. As shown in Table 5, anastomotic stenosis is often not registered in randomized controlled trials. Only two studies mention anastomotic stenosis, and show no significant differences between chemoradiation with subsequent surgery vs. surgery alone. The incidence of anastomotic stenosis in this study is relatively high; however, it is in concordance with a study in which the incidence of anastomotic stricture was 44% in patients with an end-to-end anastomosis [10].

Anastomotic leakage was not a predictor for anastomotic stenosis in our study. This is not supported by other studies in which anastomotic leakage was found to be an independent predictor for anastomotic stenosis [11,12,18]. This difference might be a result of the relatively small number of patients and the relatively high anastomotic leakage rate identified in the current study.

Other factors besides chemoradiation that might influence anastomotic problems are, according to the literature, type of resection (transthoracic vs. transhiatal) and location of anastomosis (thoracic vs. cervical) [6,10,17]. In the studied RCTs, both types of surgery have been often used and anastomotic location was mostly cervical or not mentioned at all (Table 5). Based on these heterogeneous RCTs, it is difficult to determine factors that influence anastomotic leakage and stenosis. Therefore, we investigated a homogenous patient population with the same type of resection and a strictly defined cervical anastomotic location.

This study is unique, but also has some limitations. The study population is relatively small; however, the group used here is more homogenous than others with respect to the uniform treatment and type of anastomosis. Furthermore, the determination of the future anastomotic region, despite the consultation with the consulting surgeon, may be prone to a certain degree of subjective variability. However, since we have uniformly determined the anastomotic region within a pre-defined distance from the most proximal part of the stomach, we believe that the comparison between groups is accurate enough to draw firm conclusions from the results.

Furthermore, we were not able to compensate breathing-induced organ motion. This could have influenced the calculated dose to the region of interest. However because the PTV consisted of the CTV plus a margin of 1 cm in all directions we do not think this will have a significant influence on our results. As in other studies looking at the correlation between radiation induced morbidity and anastomotic complications, the influence of day-to-day treatment variations on the dose to the region could not be quantified as we do not have CBCT imaging data of these patients, which is a clear limitation. Realising this, it is even more striking that we did not observe a correlation between dose and morbidity, as including day-to-day variations will only blur the dose distributions we want to correlate with morbidity even more. In this study we did not measure esophagitis grade after chemoradiation. Recent study in lung cancer patients showed association between higher V50 and esophagitis [34]. The number of patients with esophagitis in our study will be comparable with recent published results from the CROSS II trial with the same chemoradiation regimen. They observed that 19% of the patients developed esophagitis [16].

## Conclusions

This study demonstrates that radiation dose on the future anastomotic region did not have a significant influence on the occurrence of anastomotic leakage and stenosis in patients with esophageal cancer treated with this commonly used regimen of neoadjuvant chemoradiation followed by transhiatal esophagectomy and left cervical anastomosis. Although with a relative small study population, our study is important for clinical practice since it suggests that radiation to the future anastomotic region within a regimen of neo-adjuvant chemoradiation with a moderate total dose has no apparent negative effect of anastomotic complications.
